# circ_0039787 promotes cervical cancer cell tumorigenesis by regulation of the miR-877-5p-KRAS axis

**DOI:** 10.18632/aging.205508

**Published:** 2024-02-02

**Authors:** Xiuchao He, Jing Sun, Jing Zhang, Binbin Zhu, Lufei Jin, Jianhua Wang, Qingyan Guan

**Affiliations:** 1 Department of Radiology, The First Affiliated Hospital of Ningbo University, Ningbo, Zhejiang, China; 2 Department of Gynecology and Obstetrics, Yinzhou No. 2 Hospital of Ningbo, Ningbo, Zhejiang, China

**Keywords:** circRNAs, cervical carcinoma, circ_0039787, miR-877-5p, KRAS

## Abstract

Circular RNA (circRNA) is a novel type of RNA that plays an important role in the occurrence and development of many malignant tumors. However, the potential regulatory role and molecular mechanisms of circRNAs in cervical cancer (CC) are still not clear. Here, we explored circRNAs associated with CC from the Gene Expression Omnibus (GEO) datasets GSE113696 and GSE102686. We initially identified circ_0039787, which is derived from exons 2 to 3 of the C16orf70 gene. We observed that circ_0039787 is mainly located in the cytoplasm and is more stable than its linear counterpart, C16orf70. circ_0039787 is significantly upregulated in CC tissues and cells. In addition, functional gain and loss experiments demonstrated that circ_0039787 promotes the proliferation, migration, and invasion of CC cells *in vitro* and the growth of CC tumors *in vivo*. Mechanistically, circ_0039787 promotes CC tumor progression by competitively absorbing miR-877-5p to alleviate the inhibitory effect of miR-877-5p on Kirsten Rat Sarcoma viral oncogene homolog (KRAS) expression. Overall, our results suggest that circ_0039787 could serve as a promising diagnostic biomarker and potential therapeutic target for CC patients.

## INTRODUCTION

Cervical cancer (CC) is associated with a high mortality rate. Around 600,000 women globally are diagnosed with CC annually, resulting in approximately 300,000 deaths [1]. Currently, the primary pathogen attributed to CC is the human papillomavirus (HPV), with persistent and recurrent infections identified as the primary cause of the disease [2]. Despite the reduction in the incidence rate and mortality of CC in most regions of the world due to screening [3], patients with advanced CC continue to experience poor outcomes. Furthermore, the pathogenesis of CC remains incompletely understood. Thus, the elucidation of the pathogenic mechanisms of CC may aid in the development of novel therapeutic approaches.

CircRNAs, which feature specific closed-loop structures, are endogenous RNAs [4] found in numerous organisms and regulate a wide range of cellular and biological functions CircRNAs have been documented to play a role in human cancers, serving not only as biomarkers [5]. CircRNAs have been documented to play a role in human cancers, serving not only as biomarkers [6], but also as regulators that can either promote or inhibit cancer growth [7]. Evidence indicates that the regulation of circRNAs significantly impacts the development and progression of CC [8, 9] despite limited understanding of the underlying mechanisms that necessitate further investigation.

MicroRNAs (miRNAs) are small RNAs which typically consist of approximately 22 nucleotides. They tend to be strongly conserved and have been found to target specific cancer-associated genes, thus influencing tumor development [10, 11]. Many miRNAs are aberrantly expressed in CC tissues where they participate in multiple tumor-associated processes [12, 13]. For example, miRNA-29a blocks cell proliferation by modulating p16 methylation in CC [14] while miR-95-3p has been proposed as a biomarker for the prediction of CC prognosis as it modulates CC progression by targeting VCAM1 [15] while miR-877-5p has been reported to contribute to the modulation of CC tumorigenesis [16]. However, our current understanding of the impact of miR-877-5p in CC is still limited.

The oncogene RAS is involved in many pathways that regulate cellular proliferation and survival, and other cellular functions [17]. Mutation and activation of the three RAS genes (KRAS, HRAS, and NRAS) are recognized in about 25% of human cancers, with Kirsten Rat Sarcoma viral oncogene homolog (KRAS) playing a significant role in the development of tumors [18]. Previous research has demonstrated that miR-16 can inhibit the proliferation of CC cells by modulating the activity of KRAS However, the occurrence of KRAS mutations in CC is infrequentcorrelationremains unclear and necessitates additional [19]. However, the occurrence of KRAS mutations in CC is infrequent [20]. Therefore, the correlation between KRAS and CC remains unclear and necessitates additional investigation.

This study explored the expression profile of circRNA in CC tissues and cells by analyzing microarray datasets from the Gene Expression Omnibus (GEO). A novel circRNA, designated as circ_0039787, was identified, although its function in tumors remains unclear. circ_0039787 is derived from the exon of the C16orf70 gene. Compared with adjacent noncancerous tissues, circ_0039787 was upregulated in CC tissues. Further investigation demonstrated that circ_0039787 upregulates the expression of KRAS by competitively absorbing miR-877-5p, thereby promoting the proliferation and metastasis of CC cells. Our findings suggest that circ_0039787 may serve as a potential therapeutic target in CC.

## RESULTS

### Identification of circ_0039787 in CC

**Figure 1:**
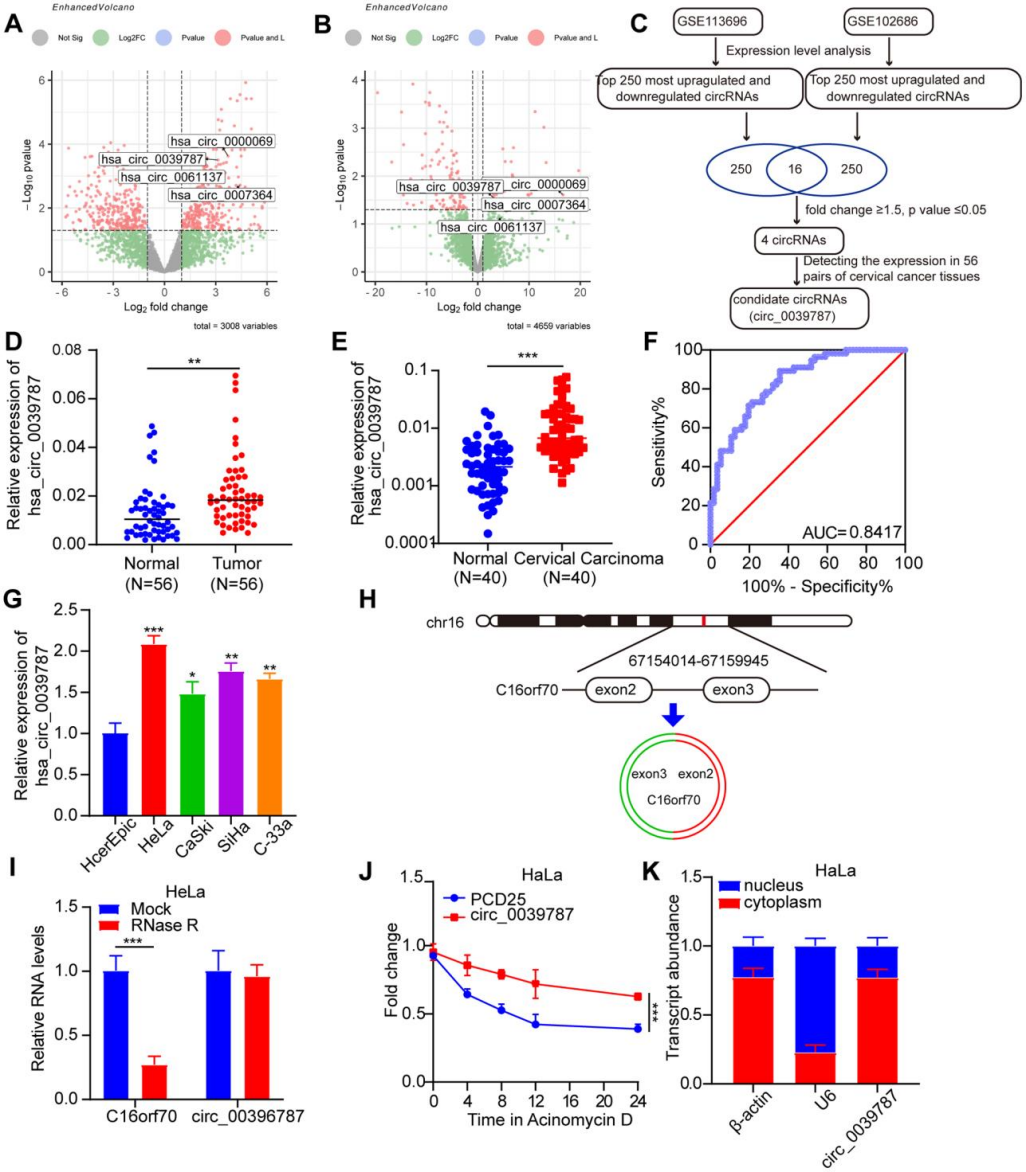
**Identification of circ_0039787 in CC.** (**A**, **B**) Differentially expressed circRNAs in CC tissues and paracancerous tissues in the GSE113696 and GSE102686 datasets. (**C**) Flow chart for screening differentially expressed circRNAs. (**D**) circ_0039787 mRNA levels in CC and paracancerous tissues, *N* = 56. (**E**) circ_0039787 expression levels in serum samples from healthy controls and CC patients, *N* = 40. (**F**) The ROC analysis for detection of CC patients from health controls using circ_0039787. (**G**) The mRNA levels of circ_0039787 were compared between CC and control cell lines. (**H**) Sanger sequencing revealed that circ_0039787 was generated by reverse-splicing exons 4, 5, and 6 of the parent gene C16orf70. (**I**) The mRNA levels of circ_0039787 and C16orf70 were measured after treatment with RNase R. (**J**) The alterations in mRNA levels of circ_0039787 and C16orf70 were observed at different time points following the addition of actinomycin D. (**K**) The mRNA levels of circ_0039787 were measured in both the nuclei and cytoplasm. ^*^P < 0.05, ^**^P< 0.01, ^***^P < 0.001.

To identify circRNAs differentially expressed between malignant and normal tissue and consequently may be associated with CC development and progression, we initially examined circRNA datasets (GSE113696 and GSE102686) available in the GEO database (Figure 1A, 1B). As shown in the volcano plot, applying the criteria of fold change >1.5 or *P* < 0.05, four differentially expressed circRNAs were identified in the GSE113696 and GSE102686 datasets (Figure 1C). The levels of circ_0000069 (Supplementary Figure 1A), circ_0039787 (Figure 1D), circ_0007364 (Supplementary Figure 1B), and circ_0061137 (Supplementary Figure 1C) in 56 pairs of CC tissues and adjacent non-tumor tissues were studied using qRT-PCR. The results revealed that compared to the adjacent non-tumor group, the expression levels of circ_0007364 and circ_0038787 were significantly upregulated in CC tissues, while there was no statistically significant difference in the expression levels of circ_0000069 and circ_0061137. Therefore, we selected circ_0039787, which showed the most prominent upregulation in CC tissues, as the candidate target. We further analyzed the relative expression levels of circ_0038787 in the serum of 40 CC patients and 40 healthy controls. The qRT-PCR results showed that compared to the healthy control group, the expression of circ_0038787 was significantly upregulated in the serum samples of CC patients (Figure 1E). The corresponding AUC value of circ_0038787 in distinguishing cancer patients from healthy controls was 0.8015 (Figure 1F). We determined the levels of circ_0039787 in the HcerEpic normal cervical epithelial cell line, as well as in the HeLa, CaSki, SiHa, and C-33A cell lines. The results showed significantly higher levels of circ_0039787 in CC cells, particularly in HeLa cells, whereas CaSki cells exhibited lower expression. Based on these findings, we selected HeLa and CaSki cell lines for our subsequent experiments (Figure 1G). circ_0039787 is derived from splicing exons 2–3 of the C16orf70 gene located on chromosome 16. The resulting circRNA consists of 167 nucleotides (Figure 1H). To assess stability, we treated circ_0039787 and linear C16orf70 mRNA with RNase R. The circular circ_0039787 exhibited higher resistance to RNase R, while the linear C16orf70 mRNA showed significant degradation (Figure 1I). Furthermore, we measured the transcriptional half-life of circ_0039787 and C16orf70 mRNA post-treatment with actinomycin D. The circ_0039787 exhibited a longer half-life compared to C16orf70 mRNA, suggesting its greater stability (Figure 1J). Subcellular localization plays a crucial role in the function of circRNA. Therefore, we quantified the distribution of circ_0039787 in the cytoplasm and nuclei of HeLa cells using qRT-PCR. The results revealed predominant cytoplasmic expression of circ_0039787 in CC cells (Figure 1K).

### circ_0039787 promotes tumorigenic behavior in CC cells

**Figure 2:**
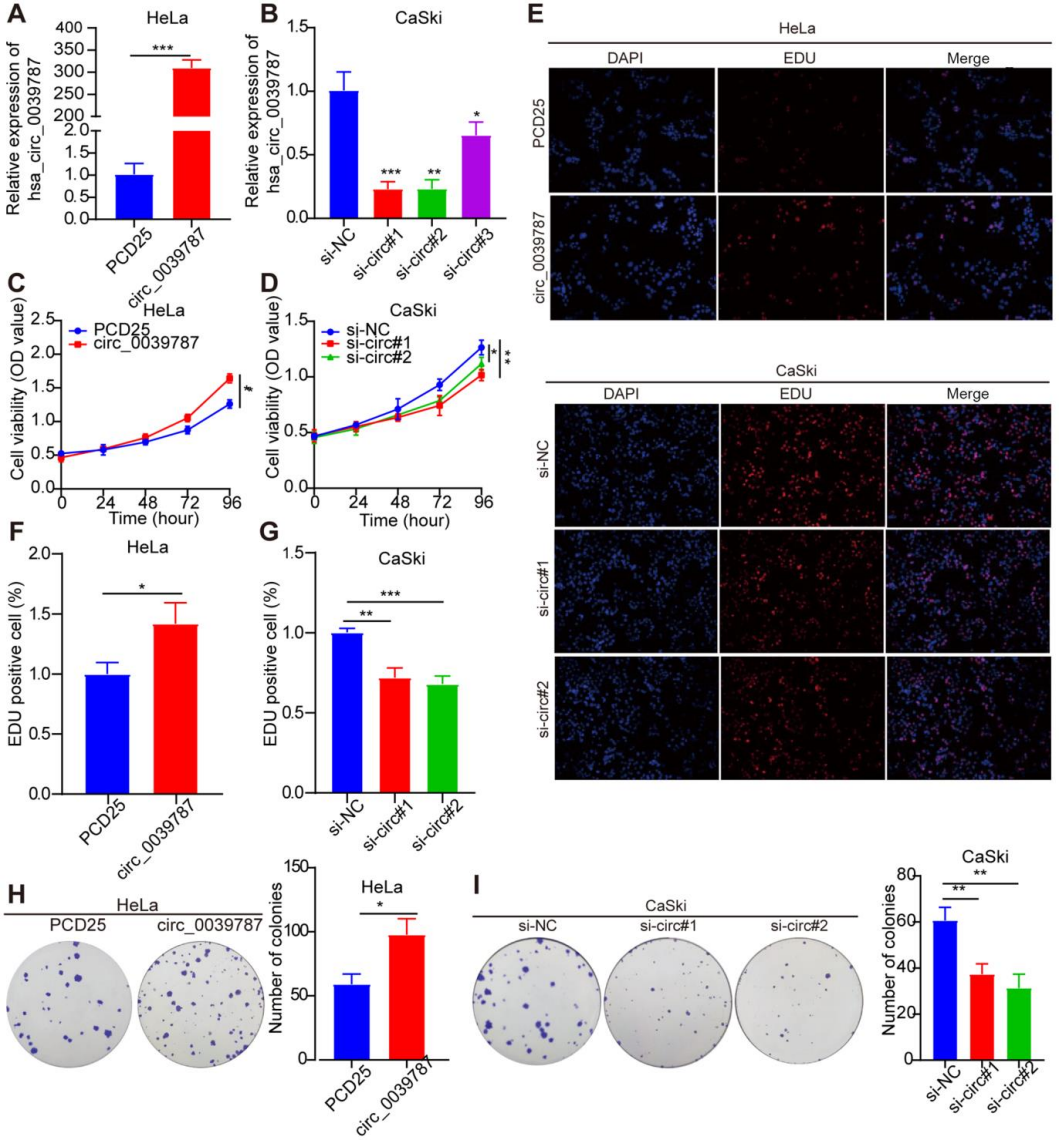
**Illustrates the promotion of tumorigenic behavior in CC cells by circ_0039787.** (**A**) circ_0039787 levels in overexpressing HeLa cells. (**B**) circ_0039787 levels in knocked down CaSki cells. (**C**, **D**) Cell proliferation changes were assessed using the CCK-8 assay following knockdown or overexpression of circ_0039787. (**E**–**I**) Cell proliferation was evaluated through EdU and colony formation assays following knockdown or overexpression of circ_0039787. ^*^P < 0.05, ^*^P < 0.01, ^***^P < 0.001.

**Figure 3:**
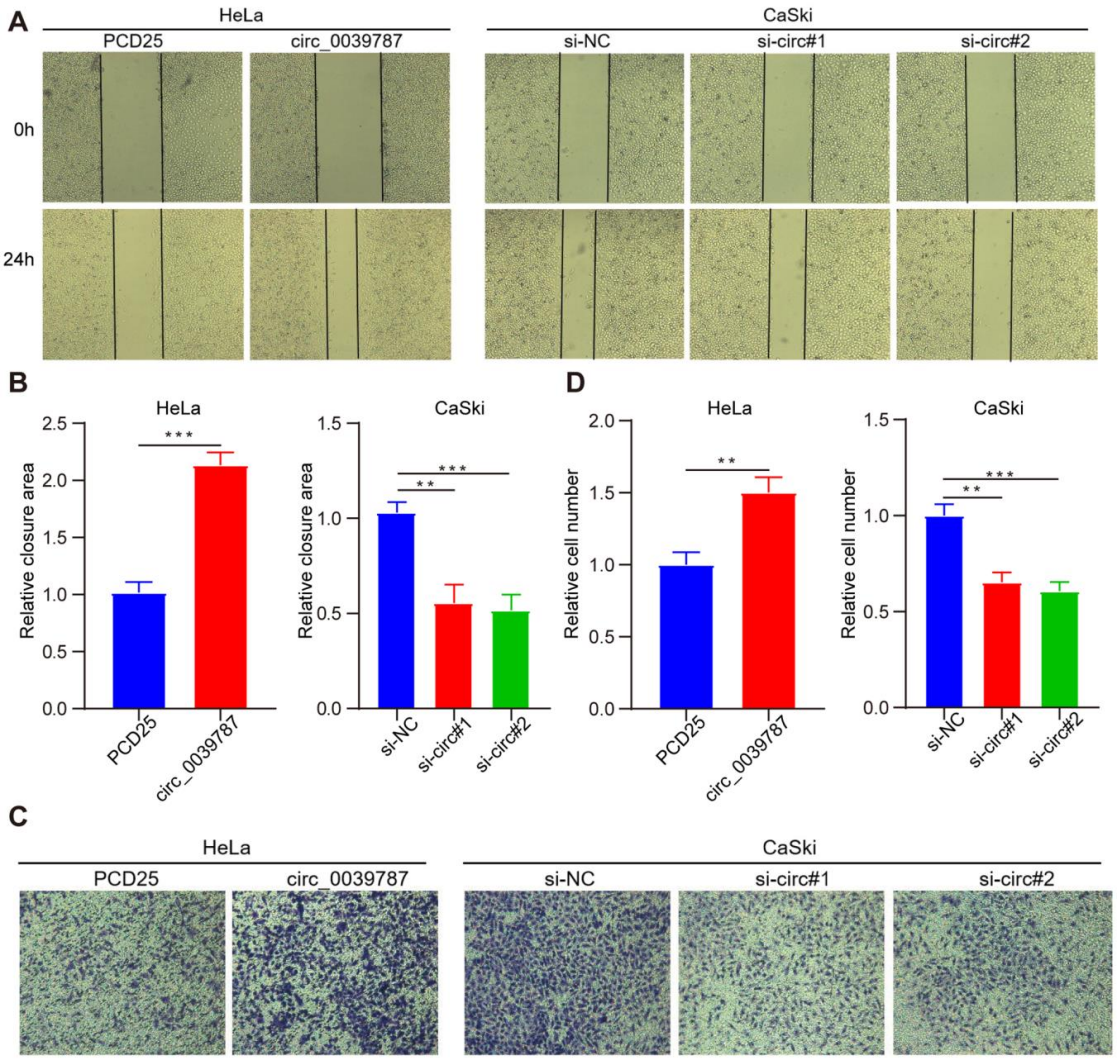
**circ_0039787 promotes the migration and invasion of CC.** (**A**, **B**) Wound-healing assays were employed to measure cell migration following knockdown or overexpression of circ_0039787. (**C**, **D**) The Transwell invasion assay was performed to evaluate cell invasion after knockdown or overexpression of circ_0039787. ^**^P < 0.01, ^***^P < 0.001.

For additional exploration of the function of circ_0039787 in CC cells, a circ_0039787 overexpression vector was constructed to establish HeLa cells overexpressing circ_0039787 (Figure 2A). circ_0039787 expression was also knocked down in CaSki cells using three siRNAs (Figure 2B). The effects of circ_0039787 on cell proliferation were examined through CCK-8, colony formation, and EdU assays. The results demonstrated that overexpression of circ_0039787 significantly enhanced the proliferation of HeLa cells (Figure 2C–2I). Conversely, the inhibition of circ_0039787 expression had the opposite effect, leading to a decrease in cell proliferation. Moreover, circ_0039787 overexpression was found to promote the invasion and migration of HeLa cells, as observed in Transwell wound-healing and invasion assays. Conversely, knockdown of circ_0039787 resulted in a reduction in HeLa cell invasion and migration capabilities (Figure 3A–3D).

### circ_0039787 negatively controls miR-877-5p in CC cells

**Figure 4:**
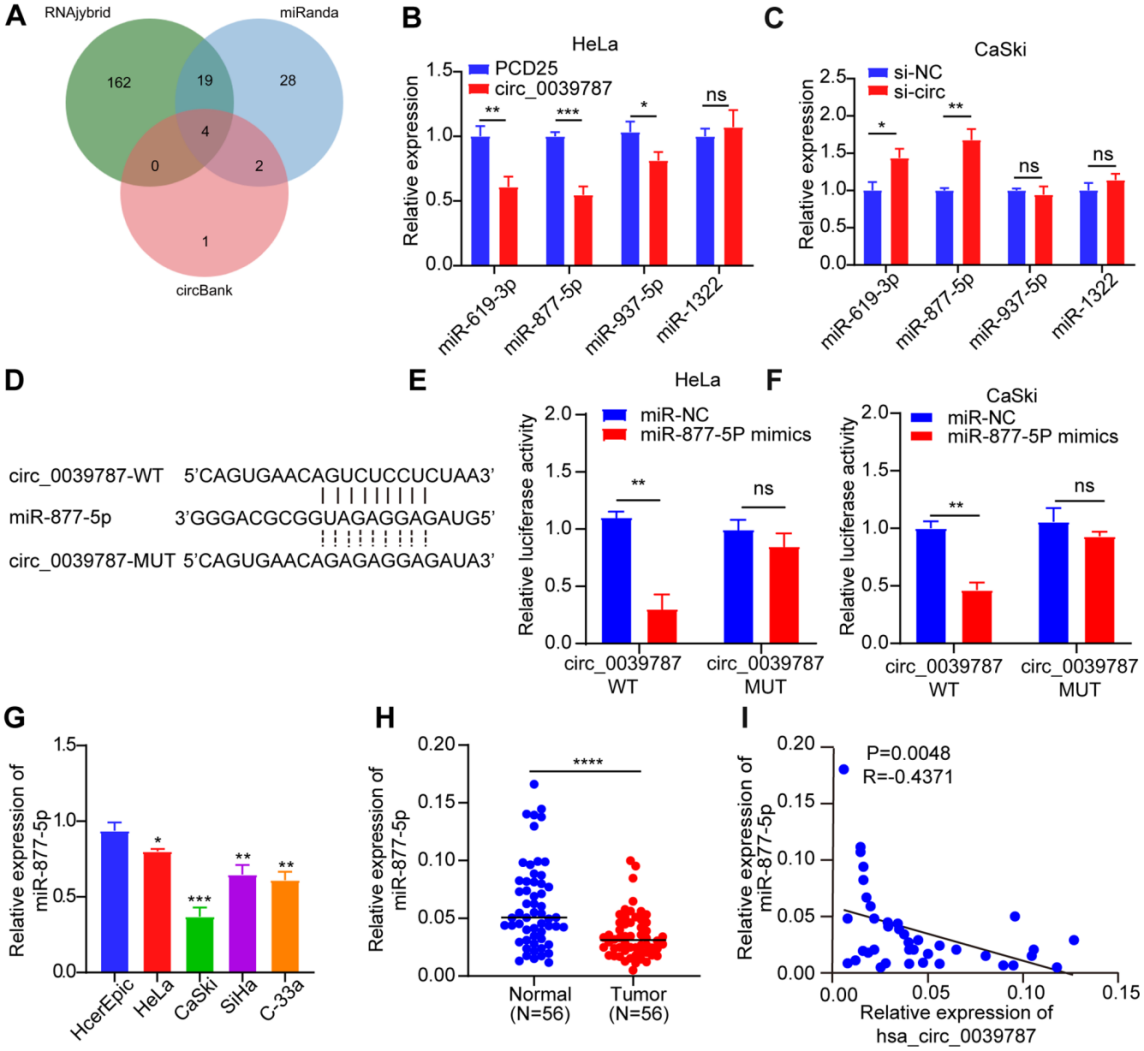
**Illustrating the negative regulation of miR-877-5p in CC cells by circ_0039787.** (**A**) Database prediction of miRNAs potentially binding to circ_0039787. (**B**, **C**) Levels of candidate miRNAs following knockdown or overexpression of circ_0039787. (**D**) Prediction of binding between miR-877-5p and circ_0039787. (**E**, **F**) Luciferase reporter assay conducted for circ_0039787-WT and circ_0039787-MUT. (**G**) Levels of miR-877-5p in CC cell lines compared to control cell lines. (**H**) Levels of miR-877-5p in CC and paracancerous tissues (*N* = 56). (**I**) Correlations between the levels of circ_0039787 and miR-877-5p in CC tissue. *P* < 0.05, ^**^P < 0.01, ^***^P < 0.001.

Several studies have provided evidence of miRNA adsorption by circRNAs, leading to the regulation of mRNA levels and translation in CC. Given the notable stability of circ_0039787 and its predominant localization in the cytoplasm of CC cells, we employed three databases (RNAhybrid, miRanda, and circBank) to predict potential target miRNAs for circ_0039787 (Figure 4A). Taking the intersection of three databases, the results show that miR-619-3p, miR-877-5p, miR-937-5p, and miR-1322 can potentially bind to circ_0039787. The expression levels of miRNAs were detected by qRT-PCR after knockdown of circ_0039787. The results show that the expression level of miR-877-5p is significantly upregulated. However, after overexpression of circ_0039787, the expression level of miR-877-5p is significantly downregulated (Figure 4B, 4C). In addition, based on the binding sites of miR-619-3p, miR-877-5p, miR-937-5p, and miR-1322 with circ_0039787, the wild-type sequence and mutant sequence of circ_0039787 were cloned into the pmirGLU vector separately. The results show that only co-transfection of miR-877-5p mimics with circ_0039787-WT vector leads to a significant decrease in luciferase activity (Figure 4D–4F). However, co-transfection of miR-619-3p, miR-937-5p, and miR-1322 mimics with circ_0039787-WT vector shows no significant change in luciferase activity (Supplementary Figure 2A–2C). These results indicate that circ_0039787 targets and binds to miR-877-5p. There was a significant downregulation of miR-877-5p in both CC tissues and cells that overexpressed circ_0039787, as observed by qRT-PCR (Figure 4G–4H). Additionally, the levels of miR-877-5p were inversely associated with circ_0039787 levels in the 56 pairs of freshly frozen CC tissues, as evidenced by Pearson correlation coefficients (Figure 4I).

### Reversal of the carcinogenic action of circ_0039787 in CC by miR-877-5p

**Figure 5:**
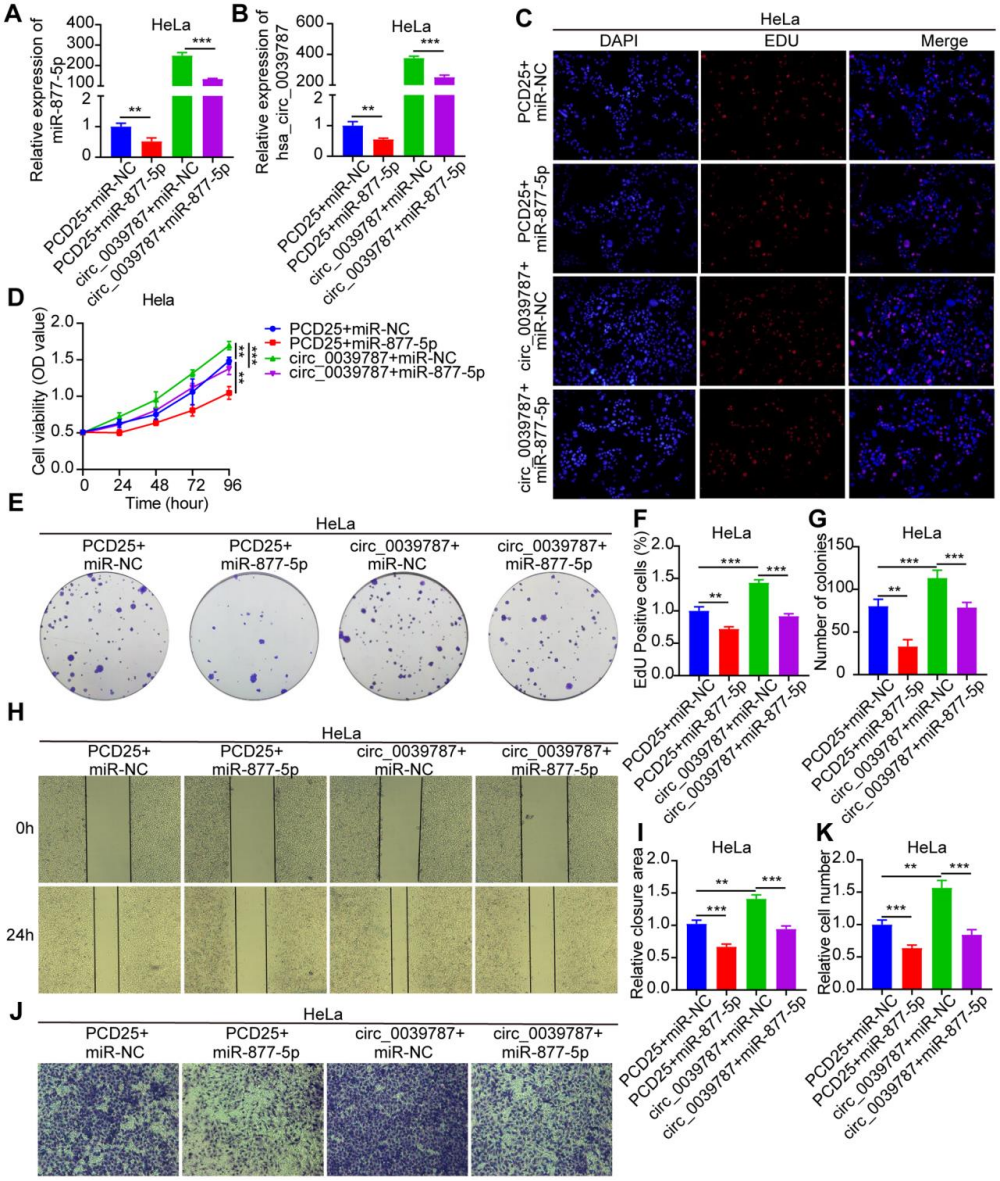
**Reversal of carcinogenic effects of circ_0039787 by miR-877-5p.** PCD25+miR-NC, PCD25+miR-877-5p mimics, PCD25-circ_0039787+miR-NC, PCD25-circ_0039787+miR-877-5p mimics in co-transfected HeLa cells. (**A**, **B**) circ_0039787 and miR-877-5p mRNA levels. (**C**–**G**) CCK-8, EdU, and colony formation assays for measuring cell proliferation. (**H**, **I**) Wound-healing assays assessing migration. (**J**, **K**) Transwell assay measuring cell invasion. *P* < 0.05, ^**^P < 0.01, ^***^P < 0.001.

A rescue experiment was designed to evaluate the regulatory role of circ_0039787 on cervical CC through miR-877-5p. HeLa cells were transfected with PCD25+miR-NC, PCD25+miR-877-5p mimics, PCD25-circ_0039787+miR-NC, or PCD25-circ_0039787+miR-877-5p mimics, and the levels of circ_0039787 and miR-877-5p were measured using qRT-PCR (Figure 5A, 5B). The biological functions of each group of cells were evaluated. The results showed that miR-877-5p could significantly reverse the promoting effects of circ_0039787 on the proliferation, migration, and invasion of CC cells (Figure 5C–5K). In conclusion, circ_0039787 promotes the malignant progression of CC(CC) by sequestering miR-877-5p.

### KRAS is a functional target of miR-877-5p

**Figure 6:**
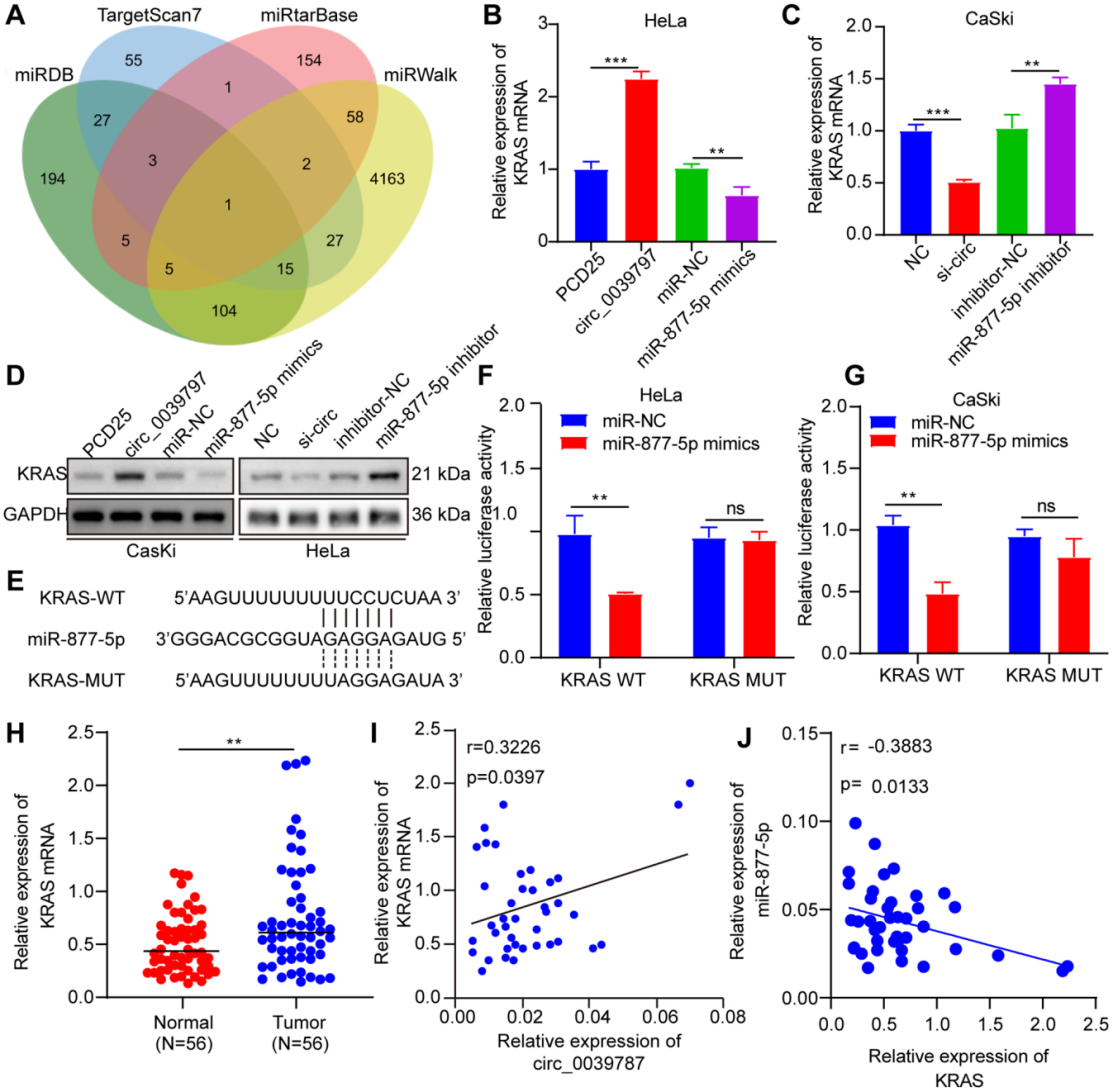
**KRAS is a functional target of miR-877-5p.** (**A**) Prediction of miR-877-5- target genes (**B**, **C**) KRAS mRNA levels. (**D**) KRAS protein levels. (**E**) Prediction of miR-877-5p and KRAS binding site. (**F**, **G**) Luciferase reporter assays of KRAS-WT and KRAS-MUT. (**H**) KRAS mRNA levels in CC and paracancerous tissues, *N* = 56. (**I**) Correlations between circ_0039787 and KRAS levels in CC tissues. (**J**) Correlations between KRAS and miR-877-5p levels in CC tissues. ^**^P < 0.01, ^***^P < 0.001.

The targets of miR-877-5p in CC were investigated using the miRDB, TargetScan, miRTarBase, and miRWalk databases (Figure 6A). Among the predicted targets by all four databases, KRAS was the only potential target. Next, the study examined how alterations in circ_003787 and miR-877-5p levels affected the expression of KRAS. Overexpression of circ_0039787 and knockdown of miR-877-5p led to significant increases in KRAS expression, whereas knockdown of circ_0039787 and overexpression of miR-877-5p resulted in reduced levels of KRAS (Figure 6B–6D). To further explore the interaction between miR-877-5p and KRAS, a luciferase reporter plasmid containing KRAS-WT and KRAS-MUT, with a mutant miR-877-5p-binding site, was constructed (Figure 6E). The luciferase activity of KRAS-WT was significantly lower when transfected with miR-877-5p mimics, but increased in CaSki cells transfected with miR-877-5p inhibitors. In contrast, the luciferase activity of KRAS-MUT mutants was unaffected by the miR-877-5p mimics or inhibitors, as well as in the negative controls, indicating a direct interaction between KRAS and miR-877-5p (Figure 6F, 6G). Furthermore, qRT-PCR analysis of tissue samples demonstrated a significant upregulation of KRAS in tumor tissues compared to normal tissues (Figure 6H). Pearson correlations revealed a negative association between KRAS levels and miR-877-5p levels in the 56 paired tumor and control tissue samples, while a positive association was observed between KRAS levels and circ_0039787 levels (Figure 6I, 6J).

### The tumorigenic effect of miR-877-5p in CC can be reversed by KRAS

**Figure 7:**
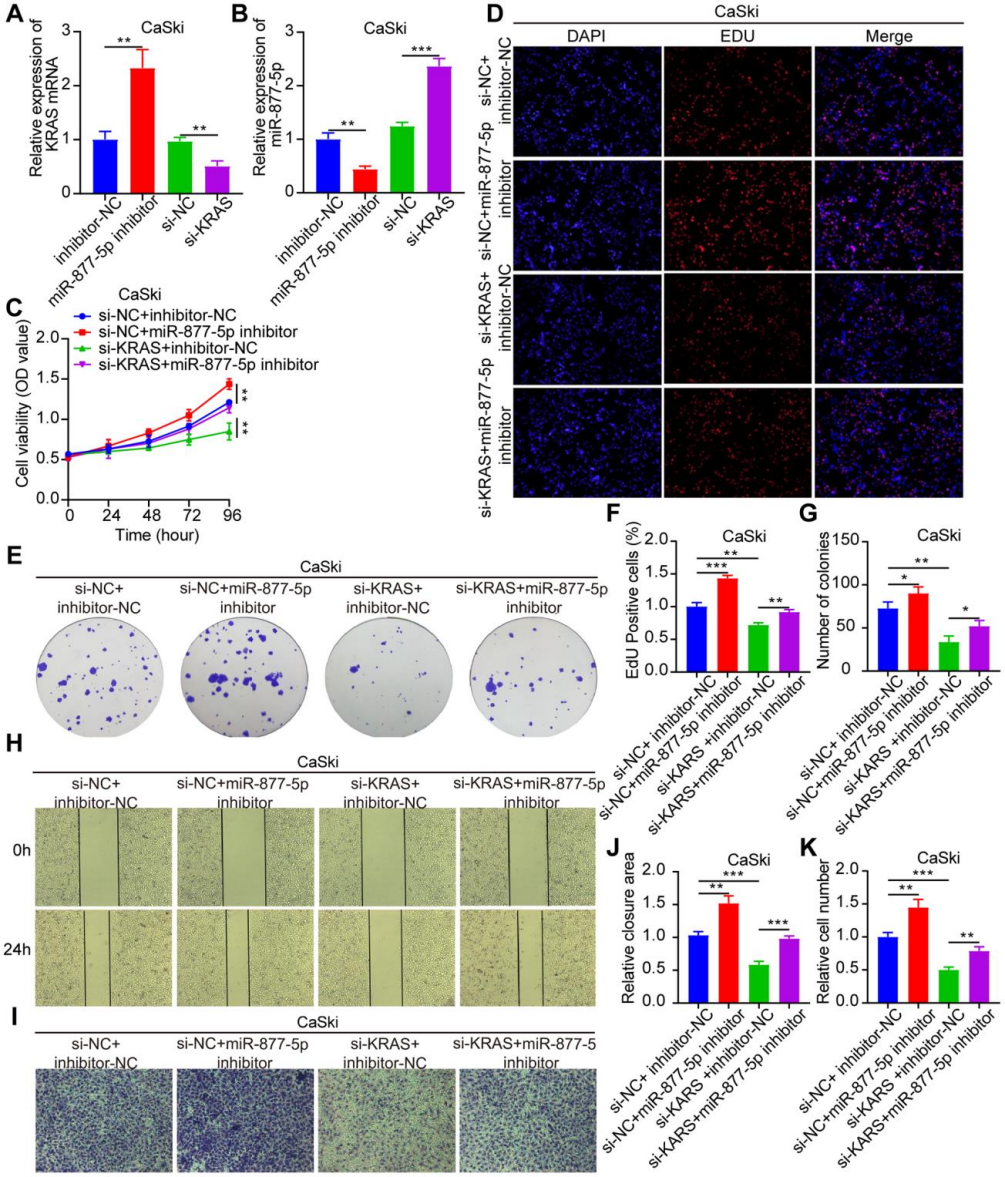
**The tumorigenic effect of miR-877-5p in CC can be reversed by KRAS.** Si-NC+inhibitor-NC, si-NC+miR-877-5p-inhibitor, si-KRAS+inhibitor-NC, and si-KRAS+miR-877-5p-inhibitor were co-transfected in CaSki cells. (**A**, **B**) qRT-PCR measurement of KRAS and miR-877-5p levels. (**C**–**G**) CCK-8, EdU, and colony formation assays measuring cell proliferation. (**H**–**K**) Wound-healing assays assessing cell migration and Transwell assays measuring cell invasion. *P* < 0.05, ^**^P < 0.01, ^***^P < 0.001.

Following this, we constructed siRNAs that specifically target KRAS. Rescue experiments were then conducted, in which CaSki cells were co-transfected with miR-877-5p inhibitors and si-KRAS (as shown in Figure 7A, 7B) to investigate their biological functions. Notably, we observed that the enhanced tumorigenic behavior seen in CC cells as a result of miR-877-5p inhibitors was effectively reversed when KRAS was present at low levels (Figure 7C–7K). These findings strongly suggest that the oncogenic activity of circ_0039787 is mediated through the miR-877-5p/KRAS axis.

### circ_0039787 prevents the body’s transplanted cancer cells from growing

**Figure 8:**
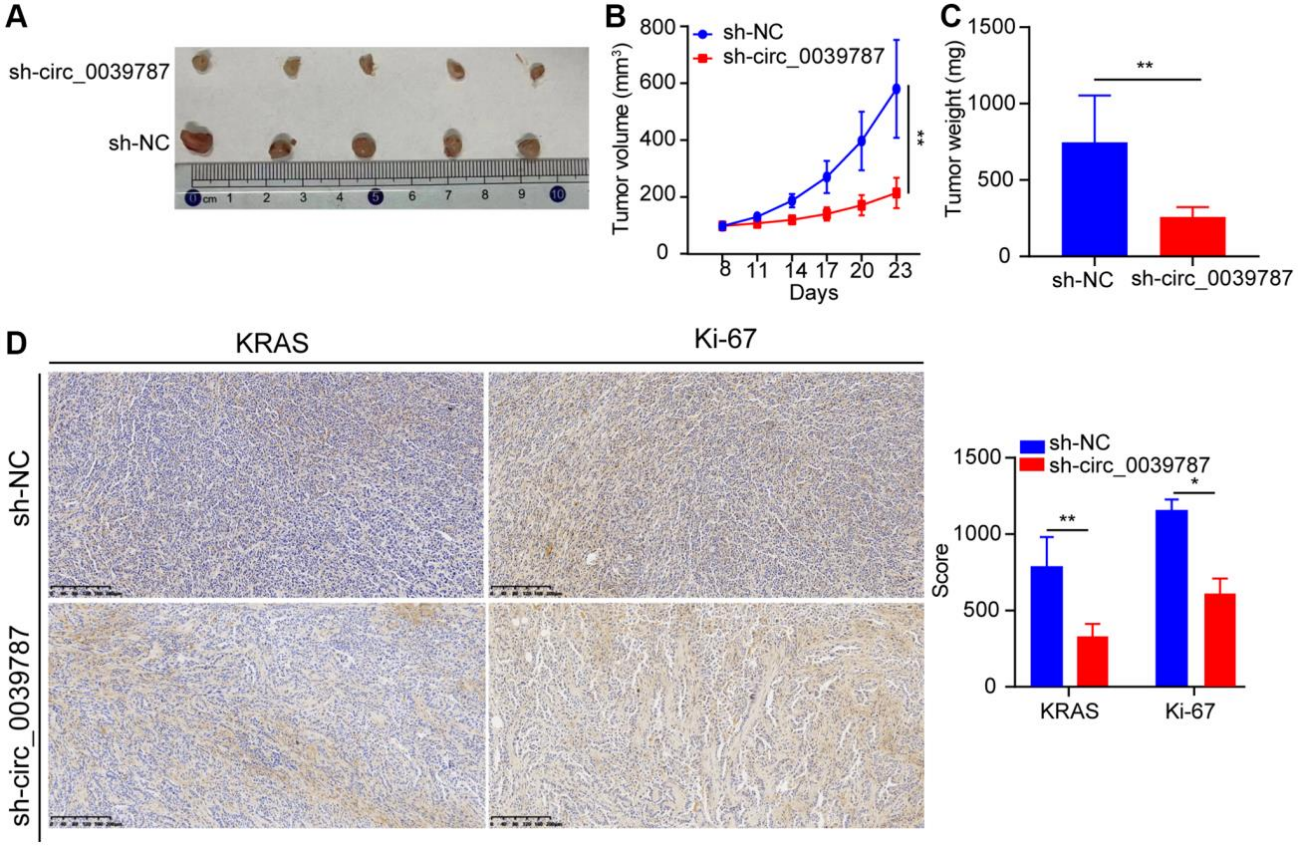
**circ_0039787 prevents the body's transplanted cancer cells from growing.** (**A**) Subcutaneous tumor development in rodents. (**B**) Volume development trajectory of subcutaneous tumors in a mouse model. (**C**) Representative images of subcutaneous tumor formation in nude mice. (**D**) Immunohistochemistry analysis of KRAS and Ki-67 expression in tumor samples. *P* < 0.05, ^**^P < 0.01.

Finally, we examined the regulatory impact of circ_0039787 on the organism using a nude mouse xenograft model. To do this, we subcutaneously administered sh-NC and sh-circ0039787 cells to nude mice and observed tumor development for a period of four weeks. It was observed that mice injected with stable circ_0039787 knockdown cells displayed a significant reduction in both tumor volume and weight compared to the control group (administered with an empty carrier) (Figure 8A–8C). Additionally, when comparing tumor samples from the sh-circ_0039787 group to control mice, a notable decrease in the expression of KRAS and Ki-67 was observed (Figure 8D).

## DISCUSSION

CC is one of the malignant tumors with a high incidence and mortality rate among women in developing countries. Surgery, radiation therapy, and chemotherapy are not ideal in terms of efficacy for patients with advanced CC, and there is an urgent need to find new treatment methods [21]. circRNA is a stable RNA molecule that usually acts as a miRNA sponge to regulate gene expression and participate in various pathological reactions [22]. It can serve as a biomarker for tumor diagnosis and prognosis evaluation, as well as a potential therapeutic target.

In our study, we explored circRNAs associated with CC using the GEO datasets GSE113696 and GSE102686. We identified several circRNAs that exhibited significant differential expression and validated them in CC tissue samples. We found that circ_0039787 was significantly upregulated in CC tissues, serum, and cell lines. ROC curve analysis demonstrated the diagnostic value of circ_0039787 in CC, suggesting its potential as a promising early diagnostic biomarker. Subsequent functional experiments revealed that knockdown of circ_0039787 significantly inhibited the proliferation, migration, and invasion of CC cells, while overexpression of circ_0039787 significantly promoted these processes, indicating its oncogenic function. Importantly, *in vivo* validation demonstrated that knockdown of circ_0039787 significantly suppressed the proliferative capacity of CC. Therefore, we hypothesize that circ_0039787 plays a critical role in the malignant progression of CC.

In circRNA research, the miRNA sponge mechanism has been an important approach to explore the biological functions of circRNAs [23]. Hansen et al. first discovered that circRNAs can act as miRNA sponges, and demonstrated that ciRS-7 functions as a miRNA sponge [24]. Since then, numerous circRNAs with miRNA sponge functionality have been identified in diseases. For example, circ_102002 is highly expressed in papillary thyroid carcinoma and promotes cell migration and EMT in thyroid carcinoma cells through the miR-488-3p/HAS2 axis [25]. CircRNA_400029 promotes the malignant biological behavior of CC by regulating the miR-1285-3p/TLN1 axis and inhibiting apoptosis, and downregulation of CircRNA_400029 can suppress tumor growth in nude mice [26]. In this study, we predicted the potential miRNA binding partners for circ_0039787 using target prediction databases (miRanda, circbank, and RNAjybrid). We identified a potential binding site between miR-877-5p and circ_0039787, and through experiments, we confirmed the targeting relationship between miR-877-5p and circ_0039787. Furthermore, the expression level of miR-877-5p in CC tissues was found to be negatively correlated with the expression of circ_0039787. Rescue experiments showed that overexpression of miR-877-5p partially restored the promoting effects of circ_0039787 on cell proliferation, migration, and invasion. From the aforementioned studies, it can be inferred that circ_0039787 exerts its biological functions by sequestering miR-877-5p.

We performed predictive analysis on the downstream target genes regulated by miR-877-5p and found that miR-877-5p can target the oncogene KRAS, exerting a tumor-suppressive function in CC. Previous studies have shown that miR-877-5p is significantly downregulated in various tumors and targets the 3′UTR of oncogenes to inhibit their expression, thereby inhibiting tumor progression. For example, in gastric cancer, miR-877-5p is downregulated in gastric cancer tissues and inhibits the growth and cell cycle progression of gastric cancer cells and promotes apoptosis through targeting FOXM1 [27]. In prostate cancer, the expression level of miR-877-5p is lower in cancer adjacent tissues, and it inhibits the malignant progression of prostate cancer through targeting SSFA2 [28]. Furthermore, in liver cancer tissues and cells, miR-877-5p is downregulated and it inhibits the proliferation, migration, and invasion of liver cancer cells by targeting CDK14 [29]. In our experiments, we confirmed that miR-877-5p can specifically target the 3′UTR region of KRAS and regulate its expression level.

KRAS is the most common mutated gene in cancer, and KRAS mutation is a known driving factor in three of the deadliest cancers: lung cancer, colorectal cancer, and pancreatic cancer [18, 30]. KRAS mutations often occur simultaneously with tumor protein p53 in pancreatic ductal adenocarcinoma, Kelch-like ECH-associated protein 1 in non-small cell lung cancer, and PIK3CA mutation in colorectal cancer [31]. As a signaling protein, KRAS is involved in many intracellular signaling pathways that regulate processes such as tumor cell growth and angiogenesis. Our data shows a significant upregulation of KRAS expression in CC tissue compared to normal adjacent tissue, and it is significantly positively correlated with the expression level of circ_0039787. Subsequent rescue experiments also demonstrate that the miR-877-5p inhibitor restores the inhibitory effect of knockdown circRNA_0000392 on CC cell proliferation, migration, and invasion. These findings suggest that the circ_0039787/miR-877-5p/KRAS axis plays a crucial role in CC.

However, our study has several limitations. Firstly, we only established subcutaneous xenograft models and did not construct lung metastasis models. Secondly, we only detected the expression of circ_0039787 in serum samples from 40 CC patients, which is a relatively small sample size. Lastly, we only focused on the roles of circ_0039787 in CC cell proliferation and metastasis. Further detailed research is necessary to explore the effects of circ_0039787 on other malignant biological behaviors, including drug resistance, angiogenesis, and immune evasion.

## CONCLUSION

In conclusion, our study demonstrates that circ_0039787 promotes the progression and metastasis of CC through the miR-877-5p/KRAS axis. Our findings elucidate a novel regulatory network that may provide new insights into the identification of potential biomarkers or therapeutic targets in CC.

## MATERIALS AND METHODS

### Tissues and cells

A total of 56 CC patients provided CC tissues who were treated at the Affiliated Hospital of Ningbo University School of Medicine between January 2021 and October 2022. None of the patients had undergone any treatment prior to biopsy or surgery. Following surgery, the resected CC tissue and the matched adjacent tissue samples were immediately frozen in liquid nitrogen and stored at −80°C until analysis. The study protocol was approved by the Ethics Committee of the Affiliated Hospital of Ningbo University School of Medicine. Written informed consent was obtained from all patients. Clinical and histopathological data were collected from medical records and pathology reports.

The human HeLa CC cell line was cultured in DMEM (Gibco, USA), and the human CC CaSki cell line was grown in RPMI-1640 medium (Gibco). The media were supplemented with 10% fetal bovine serum (Gibco) and 1% penicillin/streptomycin (PS). The cells were maintained at 37°C in a 5% CO_2_ incubator.

### RNA extraction and qRT-PCR

Total RNA was extracted from cells and tissues using TRIzol reagent, following the manufacturer’s instructions. For circRNA and mRNA analysis, the total RNA was reverse-transcribed to cDNA using the PrimeScript RT kit (Jima, China) and amplified with SYBR Green Premix Ex Taq II (Jima). For miRNA analysis, the total RNA was reverse-transcribed to cDNA using the microRNA reverse-transcription kit (Novoprotein, China) and amplified with qPCR Master Mix (Novoprotein). The internal controls used were β-Actin, GAPDH, and U6. Gene expression was normalized to these controls using the 2^−ΔΔCT^ method. The primer sequences can be found in Table 1.

**Table 1:** Primer and siRNA sequences used in this study.

**Name**	**Sequences**
circ_0039787 primer	Forward: 5′-GACTCAGGACGGGATCAAAC-3′
	Reverse: 5′-GAACCTGGACGTTTTTGATGA-3′
β-actin primer	Forward: 5′-ACAGGCATCGTGATGGATTCT-3′
	Reverse: 5′-CAGCAGTGGTGGTGAAGTTAT-3′
U6 snRNA primer	Forward: 5′-CTCGCTTCGGCAGCACA-3′
	Reverse: 5′-AACGCTTCACGAATTTGCGT-3′
miR-877-5p primer	Forward: 5′-GTAGAGGAGATGGCGCAGGG-3′
	Reverse: 5′-CAGTGCGTGTCGTGGAGT-3′
KRAS primer	Forward: 5′-GACTCTGAAGATGTACCTATGGTCCTA-3′
	Reverse: 5′-CATCATCAACACCCTGTCTTGTC-3′
si-NC	Sense: 5′-UUCUCCGAACGUGUCACGUTT-3′
	Antisense: 5′-ACGUGACACGUUCGGAGAATT-3′
si-circ_0039787#1	Sense: 5′-UCAGAGACUUAAGGAAUGCCUTT-3′
	Antisense: 5′-AGGCAUUCCUUAAGUCUCUGATT-3′
si-circ_0039787#2	Sense: 5′-AUCAGAGACUUAAGGAAUGCCTT-3′
	Antisense: 5′-GGCAUUCCUUAAGUCUCUGATTT-3′
si-circ_0039787#3	Sense: 5′-GACUUAAGGAAUGCCTCUGGCTT-3′
	Antisense: 5′-GCCAGAGGCAUUCCUUAAGUCTT-3′

### Cell transfection

PCD25 siRNA transfection was performed using Lipofectamine 2000 (Invitrogen, USA) according to the provided instructions. After 48 hours, total RNA and protein were extracted. The siRNA sequences are listed in Table 1.

### RNase R treatment

2 μg of total RNA was incubated with 5 U/μg of RNase R (Epicenter Technologies, USA) at 37°C for 30 minutes. Untreated controls were used. qRT-PCR was used to evaluate the levels of circ_0039787 and its parental gene C16orf70.

### Actinomycin D assays

Cells were cultured in 6-well plates at a density of 4 × 10^5^ cells/well and grown for 24 hours. After that, Actinomycin D (2 μg/ml; Sigma, USA) was added and the total RNA was extracted at specified time intervals. circ_0039787 and C16orf70 levels were determined using qRT-PCR.

### Nucleocytoplasmic fractionation

RNA was isolated from the nuclei and cytoplasm using a Cytoplasmic and Nuclear RNA Purification Kit (Norgen Biotek, Canada). The levels of circ_0039787 and C16orf70 were quantified by qRT-PCR, with U6 and β-actin serving as internal references for the nuclei and cytoplasm, respectively.

### CCK-8 assay

Cell proliferation was assessed using a CCK-8 kit (Yeasen, China). Cells were seeded in 96-well plates at a density of 5 × 10^3^ cells per well and incubated for 0, 24, 48, 72, and 96 hours. Afterwards, 10 μL of CCK-8 solution was added to each well and incubated for 2 hours. The absorbances at 450 nm were measured using a spectrophotometer.

### EdU assay

The BeyoClick™ EdU-555 Cell Proliferation Kit (Beyotime, China) was utilized following the instructions provided. Following 2-hour incubation with EdU, the cells were fixed with a 4% paraformaldehyde solution, permeabilized, and then stained with Alexa Fluor 555 and Hoechst 3342. Subsequently, the cells were examined using an inverted fluorescence microscope to evaluate the presence of EdU.

### Colony formation assay

Cells were seeded in 6-well plates at a density of 1 × 10^3^ cells per well and cultured for a period of 14 days. Following the aforementioned fixation procedure, the cells were subsequently stained with a 1% crystal violet solution for 15 minutes, after which they were counted.

### Wound-healing assay

The cells were cultivated in 6-well plates until reaching 90% confluence. A 200-μL pipette tip was then employed to create uniformly sized scratches in the monolayer, and the cells were subsequently visualized using a microscope. Following a 24-hour and 48-hour incubation period in serum-free medium, the cells were once again imaged, and the widths of the wounds were documented.

### Transwell invasion assay

A total of 200 μl of cell suspension in serum-free medium was carefully introduced into the upper chamber of a Transwell apparatus (Corning, USA), accompanied by the addition of 750 μl of medium containing 10% FBS into the lower chamber. Following a 48-hour incubation period, the cells residing in the upper chamber were gently scraped off using a cotton swab. The migrated cells on the lower side of the membrane were subsequently fixed, stained using the previously mentioned protocol, and the cell numbers were determined using ImageJ software.

### Western blotting

The cells were lysed in RIPA buffer (Solarbio, China) supplemented with 1% PMSF to extract the total protein. Subsequently, equivalent protein concentrations were separated by 10% SDS-PAGE, transferred onto PVDF membranes, and then subjected to a 30-minute blockage step with fat-free milk. The membranes were then incubated with the primary anti-KRAS antibody (Abcam, UK) at 4°C for 12 hours, followed by the secondary antibody at room temperature for 1 hour. Visualization of protein bands was achieved using an Electrochemiluminescence Kit (Advansta, China). GAPDH (Abcam) served as the loading control.

### Luciferase reporter assay

Synthetic sequences that bound to miR-877-5p within the circ_0039787 region, as well as their corresponding mutant sequence, were inserted into the luciferase reporter vector pmirGLU, denoted as circ_0039787-WT and circ_0039787-MUT, respectively. These plasmids were co-transfected with miR-877-5p mimics or a simulated negative control (NC). The relative luciferase activities were assessed using a Pierce Renilla-Firefly Luciferase Dual Detection Kit (Thermo Fisher Scientific, USA) according to the provided instructions.

### *In vivo* tumorigenesis assay

Four-week-old male nude mice were obtained from the Beijing Weitong Lihua Corporation (Shanghai, China). Prior to the experiment, the mice were acclimatized and allowed to familiarize themselves with the animal room at Ningbo University Medical School. The sh-NC and sh-circ 0039787 overexpressed cells were cultured and prepared before being injected into the axillary regions of the nude mice at a concentration of 1 × 10^6^ cells. The mice were weighed prior to injection. Every seven days, the tumor’s shortest (a) and longest (b) dimensions were measured using a vernier caliper. Tumor volume was calculated using the formula: Tumor volume = 1/2ab^2^. After a period of four weeks, the nude mice were euthanized, and the tumors were documented and examined.

### Statistical analysis

Data were analyzed using GraphPad Prism 9 software (GraphPad Software, USA). The data were expressed as the means ± standard deviation (SD) and were obtained from at least three independent experiments. Statistical differences between groups were determined using *t*-tests, with a significance level of *P* < 0.05.

### Data availability statement

The data used to support the findings of this study are available from the corresponding author upon request.

## Supplementary Materials

Supplementary Figures
