# Supplementary material for: circ_0039787 promotes cervical cancer cell tumorigenesis by regulation of the miR-877-5p-KRAS axis
Source: Aging (Albany NY). 2024 Feb 2;16(3):2736–52. doi: 10.18632/aging.205508 (PMC10911348; doi:10.18632/aging.205508)
Supplement: Supplementary Figures [file aging-16-205508-s001.pdf]

SUPPLEMENTARY FIGURES

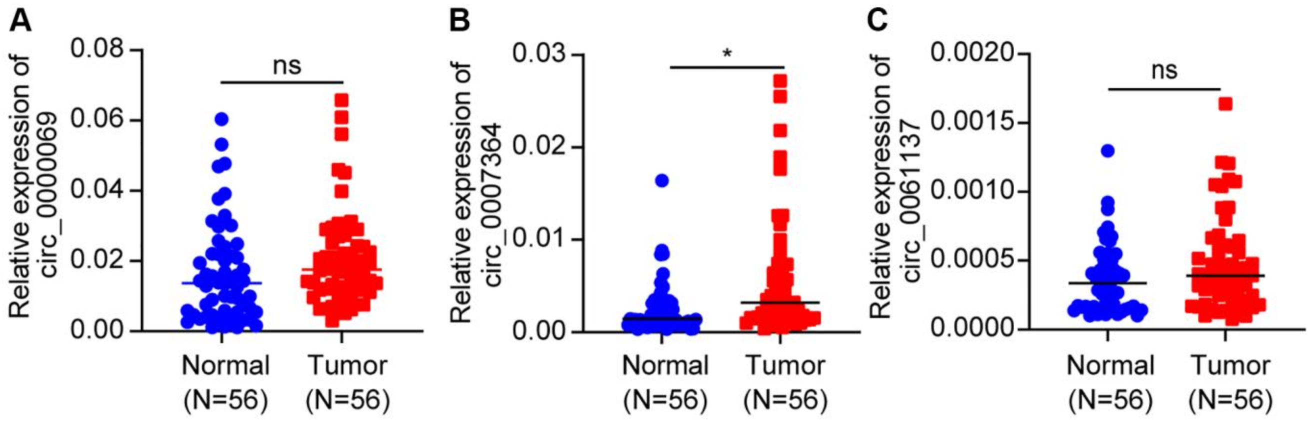

Supplementary Figure 1. Identification of circ_0039787 in CC. (**A**) circ_0000069 mRNA levels in CC and paracancerous tissues, *N* = 56. (**B**) circ_0007364 mRNA levels in CC and paracancerous tissues, *N* = 56. (**C**) circ_0061137 mRNA levels in CC and paracancerous tissues, *N* = 56.

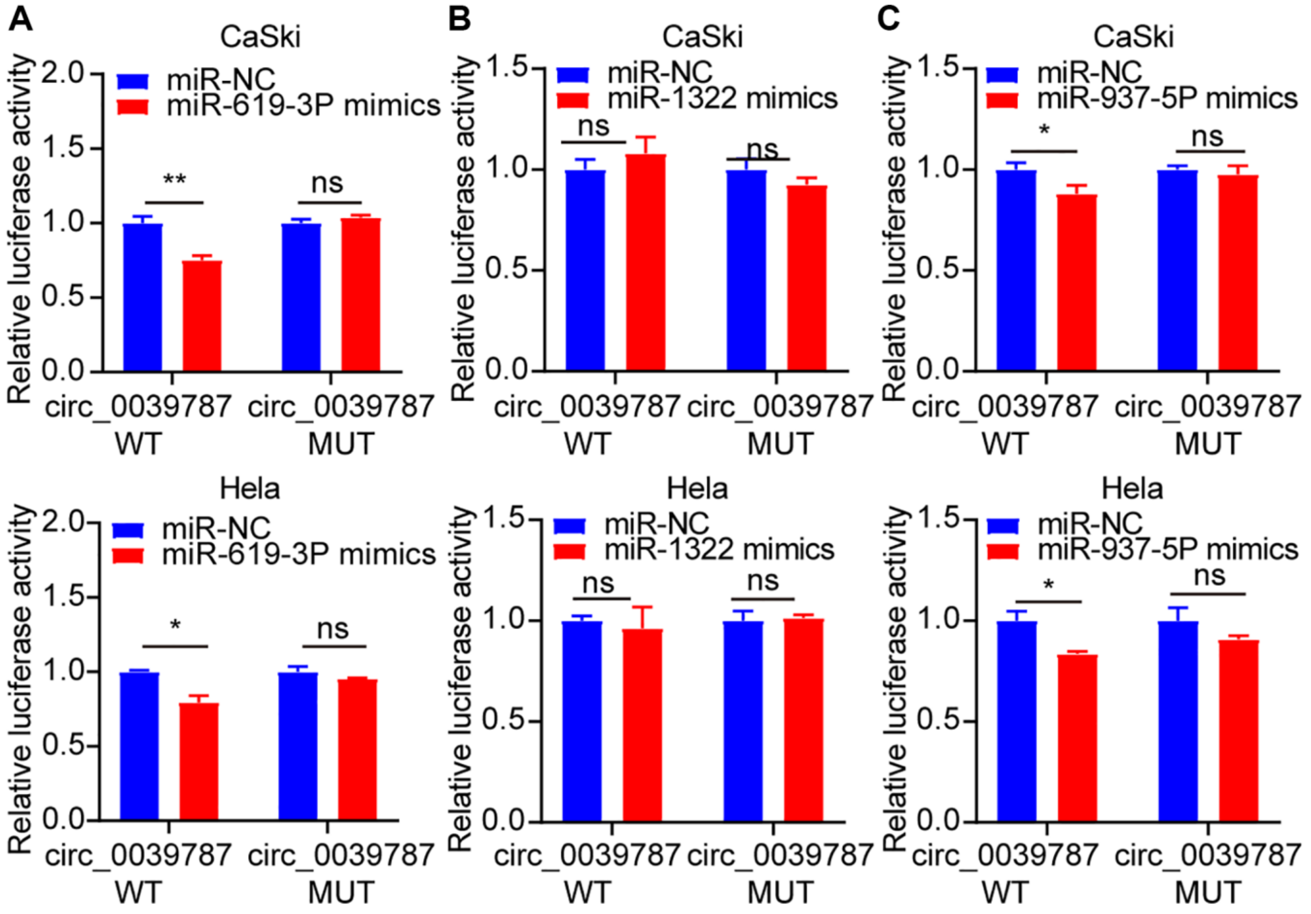

Supplementary Figure 2. Illustrates the negative regulation of miR-877-5p in CC cells by circ_0039787. (**A**–**C**) Luciferase reporter assay conducted for circ_0039787-WT and circ_0039787-MUT.
